# Phase I/II trial of Durvalumab plus Tremelimumab and stereotactic body radiotherapy for metastatic head and neck carcinoma

**DOI:** 10.1186/s12885-019-5266-4

**Published:** 2019-01-14

**Authors:** Houda Bahig, Francine Aubin, John Stagg, Olguta Gologan, Olivier Ballivy, Eric Bissada, Felix-Phuc Nguyen-Tan, Denis Soulières, Louis Guertin, Edith Filion, Apostolos Christopoulos, Louise Lambert, Mustapha Tehfe, Tareck Ayad, Danielle Charpentier, Rahima Jamal, Philip Wong

**Affiliations:** 1 Department of Radiation Oncology, Centre Hospitalier de l’Université de Montréal, 1051 Sanguinet Street, Montreal, QC H2X 3E4 Canada; 2 Department of Medical Oncology, Centre Hospitalier de l’Université de Montréal, Montreal, Canada; 3 Centre de Recherche du Centre Hospitalier de l’Université de Montréal, Montréal, QC Canada; 4 Pathology Department, Centre Hospitalier de l’Université de Montréal, Montreal, Canada; 5 Department of Otorhinolaryngology, Centre Hospitalier de l’Université de Montréal, Montreal, Canada

**Keywords:** Head and neck cancer, Metastatic, Immunotherapy, SBRT, Durvalumab, Tremelimumab

## Abstract

**Background:**

The efficacy of immunotherapy targeting the PD-1/PD-L1 pathway has previously been demonstrated in metastatic head and neck squamous cell carcinoma (HNSCC). Stereotactic Body Radiotherapy (SBRT) aims at ablating metastatic lesions and may play a synergistic role with immunotherapy. The purpose of this study is to assess the safety and efficacy of triple treatment combination (TTC) consisting of the administration of durvalumab and tremelimumab in combination with SBRT in metastatic HNSCC.

**Method:**

This is a phase I/II single arm study that will include 35 patients with 2–10 extracranial metastatic lesions. Patients will receive durvalumab (1500 mg IV every 4 weeks (Q4W)) and tremelimumab (75 mg IV Q4W for a total of 4 doses) until progression, unacceptable toxicity or patient withdrawal. SBRT to 2–5 metastases will be administered between cycles 2 and 3 of immunotherapy. The safety of the treatment combination will be evaluated through assessment of TTC-related toxicities, defined as grade 3–5 toxicities based on Common Terminology Criteria for Adverse Events (v 4.03), occurring within 6 weeks from SBRT start, and that are definitely, probably or possibly related to the combination of all treatments. We hypothesize that dual targeting of PD-L1 and CTLA-4 pathways combined with SBRT will lead to < 35% grade 3–5 acute toxicities related to TTC. Progression free survival (PFS) will be the primary endpoint of the phase II portion of this study and will be assessed with radiological exams every 8 weeks using the RECIST version 1.1 criteria.

**Discussion:**

The combination of synergistic dual checkpoints inhibition along with ablative radiation may significantly potentiate the local and systemic disease control. This study constitutes the first clinical trial combining effects of SBRT with dual checkpoint blockade with durvalumab and tremelimumab in the treatment of metastatic HNSCC. If positive, this study would lead to a phase III trial testing this treatment combination against standard of care in metastatic HNSCC.

**Trial registration:**

NCT03283605. Registration date: September 14, 2017; version 1.

## Background

### Metastatic head and neck cancer

Each year, up to 60,000 new cases of head and neck squamous cell cancer (HNSCC) are diagnosed in the United States [1]. The prevalence of distant metastasis at diagnosis varies between 4 and 26% [2–5]. Among patients without metastasis at diagnosis, up to 30% will develop distant failure [6, 7]. The prognosis of patients with distant metastasis is poor, and standard of care remains palliative chemotherapy. Palliative chemotherapy includes combinations of cetuximab, platinum and fluorouracil-based combinations associated with a median overall survival of 10 months and a response rate of 30% [8]. Alternatively, doublet platinum chemotherapy is associated with a median overall survival (OS) of 6–8 months [9]. Patients that are refractory or progress on first-line chemotherapy have limited treatment options as response rates to second-line therapies are between 3 and 13% [10].

### Local ablative therapy for oligometastatic HNSCC

Local ablation (by surgical resection or radiotherapy) of oligometastasis (defined broadly as metastatic cancer with limited burden of disease) [11], aims at achieving prolonged progression free survival (PFS), and sometimes cure [12, 13]. This model is supported by results of the phase II randomized trial by Gomez et al. [14] showing that local ablation of non-small cell lung cancer oligometastasis was associated with 3 folds increase in median PFS compared to systemic maintenance treatment (12 vs. 4 months). A meta-analysis of 13 studies including 403 patients with HNSCC that underwent surgical resection of metachronous lung metastases showed 5-year OS of 29% [15]. SBRT is a highly conformal imaged-guided radiotherapy technique allowing for delivery of an ablative dose of radiotherapy in a small number of fractions [16]. The use of SBRT as a radical approach in oligometastatic disease is attractive given its non-invasive nature, its excellent local control (above 80%) [17, 18], and its safety with < 5–10% risk of grade ≥ 3 toxicities [19–22].

### Immunotherapy in HNSCC

HNSCC tumors are highly immunogenic, with PD-L1 expression found in up to 60% of HNSCC along with elevated levels of intra-tumoral regulatory T cells infiltration [23, 24], thus making immunotherapy particularly attractive in HNSCC. Pembrolizumab and nivolumab are human IgG4 anti-PD-1 monoclonal antibodies approved as second line therapy for metastatic HNSCC by the US FDA, which respectively showed 3 months OS benefit over standard chemotherapy in platinum-refractory recurrent and metastatic HNSCC [25], and 18% response rate in recurrent or metastatic HNSCC [26, 27]. Results from Keynote 048, a randomized phase 3 study of pembrolizumab vs. cetuximab combined with platinum chemotherapy plus fluorouracil as first-line systemic therapy recurrent/metastatic HNSCC, were presented at the European Society for Medical Oncology 2018 meeting and showed that pembrolizumab was associated with significantly improved OS compared to the standard arm. Similarly, Keynote 040, a phase 3 randomized trial of pembrolizumab vs. investigator’s choice of methotrexate, docetaxel, or cetuximab, showed improved OS with pembrolizumab (8.4 vs. 6.9 months) and reduced grade 3 or worse toxicities in the treatment of recurrent or metastatic HNSCC [28].

Durvalumab (MEDI4736) is a selective human anti-PD-L1 IgG1 monoclonal antibody that blocks the interaction of PD-L1 with PD-1 and CD80. Durvalumab induced overall response rates of 11% in metastatic or recurrent HNSCC and 18% in patients with high PD-L1 expression [29]. Tremelimumab is a selective human anti-CTLA-4 IgG2 monoclonal antibody [30]. The mechanisms of action of CTLA-4 and PD-L1 pathways are non-redundant as preclinical data indicate that inhibiting both pathways have synergistic antitumor activity [31]. While anti-PD-1/PD-L1 monotherapy seems associated with a greater clinical benefit in tumors expressing PD-L1, combination with anti-CTLA-4 therapy has the potential to enhance antitumor activity of anti-PD-1/PD-L1 agents in both PD-L1 positive and PD-L1 negative tumors [32]. The combination of anti-PD-1 and anti-CTLA-4 was shown to improve PFS vs. anti-PD-1 alone in melanoma (12 months vs. 7 months) [33]. This benefit came however at the price of increased grade 3–4 toxicities (55% vs. 27%, respectively.) A multicenter phase IB study assessing the combination of durvalumab and tremelimumab in non-small cell lung cancer reported 23% objective responses, irrespective of PD-L1 status; treatment related grade 3–4 toxicities were observed in 22% of patients [34]. In HNSCC, several trials are on-going to assess the safety and efficacy durvalumab and tremelimumab vs. monotherapy in recurrent or metastatic HNSCC [35] or as first line approach in advanced HNSCC (KESTREL (NCT02551159) and EAGLE [36]).

### Combining immunotherapy and radiotherapy in head and neck cancers

Radiotherapy has been shown to induce anti-tumor immune effect in addition to cytotoxic effect [37, 38]. In fact, radiotherapy plays a role in the recruitment of T cells in the tumor microenvironment [39], secretion of cytokines, enhanced tumor antigen presentation [40, 41], and increased expression of PD-L1 in irradiated tumors [42]. In addition, induction of abscopal effect, which consists in anti-tumor response outside the radiotherapy field [42–45], has been suggested in both pre-clinical and clinical data. In mice, concomitant radiotherapy and anti-CTLA-4 antibodies induced abscopal effect [46, 47]; in addition, PD-1 blockade after completion of radiotherapy was shown to induce elimination of persistent tumors [42]. Dual checkpoint blockade (anti-CTLA-4 and anti-PD-L1) in combination with radiation has been shown to activate non-redundant immune mechanisms [38]. Single fraction radiation doses between 15 Gy and 25 Gy were shown to promote T-cell-mediated anti-tumor response at both the primary and distant metastases sites in mice model [48]. Similarly, hypofractionated regimen of 15 Gy in 1 fraction were associated with a greater tumor infiltration by immune cells compared to 15 Gy in 5 fractions regimen [49]. SBRT fractionation may therefore be advantageous for immune-stimulation and may work synergistically with immunotherapy [50]. Abscopal effect is reported in an increasing number of clinical reports, in particular in the context of the combination of immune checkpoint inhibitors and radiation [51–53].

Dual checkpoint blockade in combination with SBRT has not yet been reported in human clinical trials. Although the addition of SBRT to immunotherapy may generate higher response rates, it may increase the frequency and/or severity of toxicities. In this study, we will assess the safety and efficacy of a triple treatment combination (TTC) consisting of durvalumab, tremelilumab and SBRT in the treatment of patients with 2–10 metastasis from HNSCC.

## Methods and design

### Study design

**Fig. 1:**
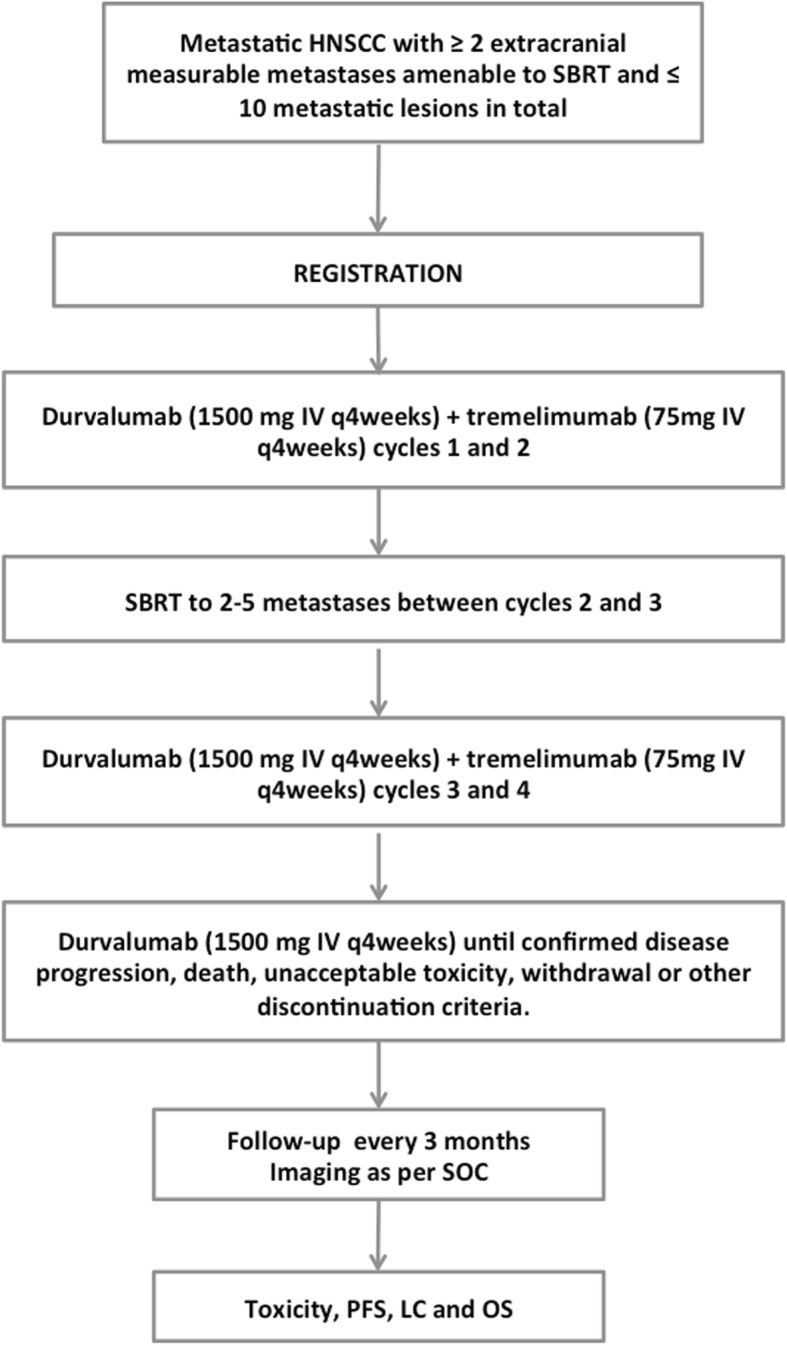
Study schema. HNSCC = head and neck squamous cell carcinoma; SBRT = stereotactic body radiotherapy; PFS = Progression free survival; LC = local control; OS = overall survival

This is a phase I/II single arm study evaluating the safety and efficacy of durvalumab, tremelimumab and SBRT combination in metastatic HNSCC. The study will include 35 patients with ≥2 extracranial measurable metastatic lesions and a maximum of 10 metastatic lesions in total, at the time of enrolment. Patients will be treated with durvalumab (1500 mg IV every 4 weeks (Q4W)) and tremelimumab (75 mg IV Q4W for a total of 4 doses) until progression, unacceptable toxicity or patient withdrawal. SBRT to 2–5 metastases will be administered between cycles 2 and 3 of immunotherapy (Fig. 1). This study is approved by the Centre de Recherche du Centre Hospitalier de l’Université de Montréal Institutional Review Board and is registered on clinicaltrials.gov (NCT03283605). Other participating academic institutions can be found on clinicaltrials.gov.

### Primary objectives

#### Phase I

To determine the safety of durvalumab and tremelimumab in combination with SBRT to 2–5 metastatic lesions by assessing rates of TTC-related serious adverse events (SAE) within 6 weeks from the start of SBRT treatments.

##### Primary endpoint

TTC-related SAE, defined as grade 3–5 toxicities based on Common Terminology Criteria for Adverse Events (CTCAE), are defined as definitely, probably, or possibly related to the combination of all 3 treatments, beyond what is expected by either treatment alone.

#### Phase II

To provide an estimate of PFS at 6-months in patients treated with durvalumab and tremelimumab in combination and SBRT.

##### Primary endpoint

PFS will be measured from the start of treatment with durvalumab and tremelimumab until the documentation of regional or distant disease progression or death due to any cause, whichever occurs first.

#### Secondary objectives

1. To assess the rate and proportion of SAE at each time point (at 3, 6, 12 and 28 weeks post-radiotherapy).
2. To estimate local control (LC) of the treatment combination.
3. To estimate median OS of the treatment combination.
4. To estimate the rate of abscopal events from the combination therapy.
5. To measure patient quality of life.
6. To correlate LC and PFS with biopsy and serum biomarkers (exploratory).
7. To correlate OS and treatment toxicity with activity tracker metrics (exploratory).

##### Secondary endpoints

LC of treated lesions will be measured from the end of SBRT treatment to date of local failure. OS will be measured from the start of treatment with durvalumab and tremelimumab to time of death. In subset of patients where at least 1 measurable lesion will not have been addressed by SBRT, abscopal effect will be estimated by comparing response rate of untreated lesion to historical response rate expected from durvalumab and tremelimumab combination alone. Quality of life will be measured in evaluable patients using the European Organization for Research and Treatment of Cancer (EORTC) Quality of Life Questionnaire Core-30 (QLQ-C30; version 3.0) and the head and neck cancer specific module EORTC QLQ-H&N35.

### Conditions for patient eligibility

1. Patient willing and able to give written informed consent
2. Patient willing and able to comply with the protocol for the duration of the study
3. ≥ 18 years of age at time of study entry
4. Body weight > 30 kg
5. Life expectancy > 24 weeks, as estimated by the treating team
6. All standard tumor-staging procedures necessary to define the baseline disease burden must be completed within 28 days to registration
7. Pathologically (histologically or cytologically) confirmed diagnosis HNSCC at a metastatic site
8. ≥ 2 extracranial measurable metastatic lesions (no brain metastases) as per RECIST v1.1, or lesions < 1 cm showing at least a 1 mm increase in size 2 consecutive imaging that amenable to SBRT.
9. ≤ 10 metastatic lesions
10. Eastern Cooperative Oncology Group/World Health Organisation (ECOG/WHO) performance status score of ≤1
11. Adequate normal organ and marrow function as defined below:
  Haemoglobin ≥9.0 g/dL
  Absolute neutrophil count (ANC ≥ 1.5 x (> 1500 per mm3)
  Platelet count ≥100 × 109/L (> 75,000 per mm3)
  Serum bilirubin ≤1.5 x institutional upper limit of normal (ULN).
  AST (SGOT)/ALT (SGPT) ≤ 2.5 x institutional upper limit of normal unless liver metastases are present, in which case it must be ≤5x ULN
  Serum creatinine CL > 40 mL/min by the Cockcroft-Gault formula
12. The following imaging workup to document metastases within 45 days prior to study registration:
  - Computed tomography of the chest, abdomen and pelvis OR whole body Positron Emission Tomography/Computed Tomography
    Patients with locoregional recurrence(s) are included only if they have evidence of distant metastasis; patients with locoregional recurrences which are symptomatic and/or potentially affect quality of life may undergo palliative radiation therapy to this region prior to enrolment on the protocol at the discretion of the treating physician. The dose and technique (conventional vs. SBRT) is at the discretion of the treating physician, with a dosimetric planning prioritization on organs at risk over tumor target coverage when in conflict. However, a minimum of 28 days must elapse before receiving protocol treatment.
13. Serum pregnancy test for female pre-menopausal patients
14. Patients who have received prior anti-PD-1, anti PD-L1 or anti CTLA-4, including durvalumab and tremelimumab if the following are fulfilled:
  Must not have experienced a toxicity that led to permanent discontinuation of prior immunotherapy.
  All adverse events (AE) of prior immunotherapy must have completely resolved or returned to baseline prior to screening for this study.
  Must not have experienced a ≥ Grade 3 immune related AE or an immune-related neurologic or ocular AE of any grade while receiving prior immunotherapy.
  Must not have required the use of additional immunosuppression other than corticosteroids for the management of an AE and not have experienced recurrence of an AE if re-challenged.

### Conditions for patient ineligibility

1. Nasopharyngeal carcinoma
2. Concurrent enrolment in another clinical study, unless it is an observational clinical study or during the follow-up period of an interventional study
3. > 4 prior treatment lines with systemic therapy
4. Receipt of the last dose of anti-cancer therapy (chemotherapy, immunotherapy, endocrine therapy, targeted therapy, biologic therapy, tumour embolization, monoclonal antibodies) ≤ 30 days prior to the first dose of study drug
5. Any unresolved toxicity CTCAE Grade ≥ 2 from previous anticancer therapy with the exception of alopecia, vitiligo, and the laboratory values defined in the inclusion criteria
  ○ Patients with Grade ≥ 2 neuropathy will be evaluated on a case-by-case basis after consultation with the Study Physician
  ○ Patients with irreversible toxicity not reasonably expected to be exacerbated by treatment with durvalumab or tremelimumab may be included only after consultation with the Study Physician
6. Any concurrent chemotherapy, investigational product (IP), biologic, or hormonal therapy for cancer treatment. Concurrent use of hormonal therapy for non-cancer-related conditions is acceptable
7. Radiotherapy treatment to more than 30% of the bone marrow or with a wide field of radiation within 4 weeks of the first dose of study drug
8. Major surgical procedure (as defined by the Investigator) within 28 days prior to the first dose of IP. Note: Local surgery of isolated lesions for palliative intent is acceptable
9. History of allogenic organ transplantation
10. Active or prior documented autoimmune or inflammatory disorders including diverticulitis, systemic lupus erythematosus, Sarcoidosis syndrome, or Wegener syndrome are excluded. However, patients without active disease in the last 5 years may enter the trial after consultation with the study physician.
11. Uncontrolled undercurrent illness that would limit compliance with study requirement, substantially increase risk of incurring AEs or compromise the ability of the patient to give written informed consent
12. History of another primary malignancy except for
  ○ Malignancy treated with curative intent and with no known active disease ≥5 years and of low potential risk for recurrence
  ○ Adequately treated non-melanoma skin cancer or lentigo maligna without evidence of disease
  ○ Adequately treated carcinoma in situ without evidence of disease
13. Presence of brain metastases or spinal cord compression. Patients with suspected brain metastases at screening should have an MRI (preferred) or CT each preferably with IV contrast of the brain prior to study entry
14. History of active primary immunodeficiency
15. Current or prior use of immunosuppressive medication within 14 days before the first dose of durvalumab or tremelimumab. The following are exceptions to this criterion:
  ○ Intranasal, inhaled, topical steroids, or local steroid injections (eg, intra articular injection).
  ○ Systemic corticosteroids at physiologic doses not to exceed 10 mg/day of prednisone or its equivalent
  ○ Steroids as premedication for hypersensitivity reactions (eg. CT scan premedication)
16. Receipt of live attenuated vaccine within 30 days prior to the first dose of durvalumab or tremelimumab
17. Known allergy or hypersensitivity to any of the study drugs or any of the study drug excipients
18. Past medical history of interstitial lung disease (ILD), drug-induced ILD, radiation pneumonitis which required steroid treatment, or any evidence of clinically active interstitial lung disease
19. Judgment by the investigator that the patient is unsuitable to participate in the study and the patient is unlikely to comply with study procedures, restrictions and requirements
20. Female patients who are pregnant or breastfeeding or male or female patients of reproductive potential who are not willing to employ effective birth control from screening to 180 days after the last dose of durvalumab + tremelimumab combination therapy or 90 days after the last dose of durvalumab monotherapy, whichever is the longer time period.

### Intervention

#### Durvalumab + tremelimumab

**Fig. 2:**
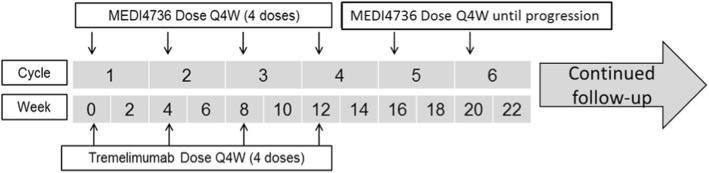
Durvalumab (MEDI4736) and tremelimumab administration schedule

Durvalumab 1500 mg plus tremelimumab 75 mg via IV infusion Q4W, starting on Week 0, for up to a maximum of 4 doses/cycles followed by durvalumab monotherapy 1500 mg via IV infusion Q4W, starting 4 weeks after the last infusion of the combination, until confirmed disease progression, death, unacceptable toxicity, withdrawal of consent, or other discontinuation criteria met (Fig. 2). Tremelimumab will be administered first; the durvalumab infusion will start at a maximum of 2 h after the end of the tremelimumab infusion.

#### SBRT

The targeting of lesions will be determined at the discretion of the treating physician. The maximum dimension receiving the prescribed SBRT dose is 4 cm. Treating physician may choose to treat a part of the target lesion’s gross tumor volume (GTV) in order to avoid exceeding the 4 cm maximum dimension limit or to reduce the chance of incurring radiation related toxicities. SBRT will be delivered during cycle 2 and completed prior to cycle 3 of durvalumab and tremelimumab combination treatment. SBRT for all metastases should be completed within 3 weeks of the first dose of SBRT. Metastases will be treated on an every other day schedule. However, a patient may receive radiation for different metastases on consecutive days.

#### Planning

Patients positioning and immobilization device will be at the discretion of the treating physician. Positioning should be stable to avoid uncontrolled movement during treatments and maintain treatment accuracy. Patient immobilization must be reliable enough to ensure that the gross tumor volume does not deviate beyond the confines of the planning treatment volume (PTV).

All patients will undergo planning CT of the region containing the treated metastasis. CT scan range will be as per standard limits used in our department for each anatomical site. Use of intravenous contrast will be required for liver metastases, but will be as per treating physician for all other sites. In addition, a 4D planning CT scan will be obtained for all metastases with potential for respiratory motion. Motion management strategies, including internal target volume technique, tracking, as well as abdominal compression, active breathing control, gating, breath hold, should be used as required. Image guided radiotherapy technique will therefore include orthogonal in-room 2D kV X-rays in cases of near-rela time robotic tracking, or volumetric imaging (kV cone beam computed tomography; MV computed tomography).

#### Dose & prescription

SBRT doses will vary based on tumor location (Table 1). Patients will receive 3 or 5 SBRT fractions. The following table details suggested dose per tumor site, as per NRG-BR001 protocol (NCT02206334):

**Table 1:** Suggested dose and number of fractions per tumor site

Site	Dose (Gy)	Fractions
Lung-peripheral	45	3
Lung-central	50	5
Mediastinal/cervical lymph node	50	5
Liver	45	3
Spinal/paraspinal	30	3
Osseous	30	3
Abdominal-pelvic metastasis (lymph node/adrenal gland)	45	3

The prescription isodose line covering 95% the PTV will generally be 80–90% but may range from 60 to 90% where the maximum dose is 100%. All dose calculations will be performed using corrections for tissue heterogeneities.

#### Target volume determination

GTV definition will be based on planning CT as well as any other standard multi-modality imaging used in the clinic. Clinical target volume and PTV definition will depend on tumor site and will be as per institutional protocols.

1. Central lung tumor will be defined as tumors within or touching the zone of the proximal bronchial tree, defined as a volume 2 cm in all directions around the proximal bronchial tree (carina, right and left main bronchi, right and left upper lobe bronchi, intermedius bronchus, right middle lobe bronchus, lingular bronchus right and left lower lobe bronchi) as well as tumors that are immediately adjacent to mediastinal or pericardial pleura (PTV touching the pleura), as per RTOG 0813.
2. Peripheral lun**g** tumors will be defined as metastases within the lungs outside of the central tumor definition above. Rib/scapular metastases within the thorax adjacent to lung parenchyma will be classified into the lung metastasis location given the similar normal tissues at risk.
3. Mediastinal/Cervical lymph nodes (LN): Mediastinal LN will include tumors arising within the anatomic space between the lungs, above the diaphragm, and below the thoracic inlet at the level of the top of the sternal notch. Cervical LN will include tumors occurring within cervical lymph node Levels I-VI and/or retropharyngeal spaces. Sternal metastases will be assigned to the mediastinal/cervical lymph node location given the similar normal tissues at risk.
4. Liver will include metastasis arising within the liver, however, as per NRG-BR001, rib metastases immediately adjacent to the liver will be assigned to the liver metastasis location.
5. Abdominal-pelvic tumors will include metastasis arising within the anatomic space defined by the diaphragm superiorly, the genitourinary diaphragm inferiorly including the peritoneal and retroperitoneal spaces, not including liver, osseous, or spinal metastases. Rib metastases adjacent to the stomach/abdominal wall will be classified into the intra-abdominal location given the similar normal tissues at risk.
6. Spinal metastases could involve any portion(s) of the vertebral spine. Rib metastases that are within 1 cm of the vertebral bodies will be classified into the spinal metastasis location given the similar normal tissues at risk.
7. Osseous lesions will be defined as any bone metastasis not included in all other site definition.

#### Critical structures

Dose limits used for organs at risk will be as per NRG-BR001 protocol, with prioritization of normal tissues over tumor coverage.

### Study assessments

Table 2 summarizes schedule of assessments during the participation to this study.

**Table 2:** Schedule of study assessments

Required investigations	Screening	Day 1 of each Cycle^1^	Treatment with SBRT (Cycle 2)	End of Cycle 2, 4, 6, 8, 10, and 12	Safety Visit(28 days after end of treatment)	Follow up^15^(Every 3 months from EOT)
Window	Within 28 days prior to registration	±3 days		±7 days	±7 days	±7 days
History and physical examination	X	X			X	X
Vital signs	X	X			X	
FFPE tumor tissue sample collection for PD-L1 assay	X				X	
Urine hCG/serum βhCGa	X					
Durvalumab		X				
Tremelimumab		X				
SBRT			X			
ECG	X	X				
ECOG Status	X	X			X	
Hematology	X	X			X	
Serum chemistry: complete clinical chemistry panel, creatinine clearance, liver enzyme panel.	X	X			X	
Urinalysis	X	X				
Thyroid function test: TSH, fT3 and fT4	X	X				
Correlative samples collection: plasma, serum, whole blood	X	X	X		X	
Coagulation parameters: PPT, APTT and INR	X					
Tumor assessments: CT head/neck, CT chest, CT abdomen/pelvis, FDG-PET, biopsy	X			X	X	As per SOC
Patient questionnaires	X	X				
Adverse events & concomitant medications review		Continuous				
Activity Tracker	Continuous					

*FFPE* Formalin-Fixed Paraffin-Embedded, *EOT* End of treatment, *SOC* standard of care

#### Data collection

The current study will be coordinated by a clinical research organization, Ozmosis Research Inc. Data will be collected centrally using electronic Case Report Forms designed and built in Medidata Rave. Data are stored within the University Health Network server in Canada. All data points will be reviewed by Ozmosis Research Data Managers. To ensure the quality of the data, site initiation will be conducted, where all components required for the conduct of the study will be evaluated prior to study opening. A risk-based monitoring visit plan was designed and at least 4 visits per year will occur to ascertain the quality of the data. One close out visit per accrual site is also planned. The monitor and trial audit is independent of the investigators and sponsor. Follow up for survival status and subsequent anti-cancer therapy will occur every 3 months from the time of treatment discontinuation. As the trial will be conducted across Canadian institutions, patient death, a public record captured for all Canadian citizens, can be collected from provincial health care databases if necessary to ensure the availability of overall survival data.

#### Biological sampling procedures

All patients entered into the study must provide blood samples, consent to the use of previously obtained paraffin-embedded tissues, and their clinical data for collection and translational studies. Paraffin-embedded tissues from any prior biopsies or resections will also be collected for correlative sciences.

#### Assessment of safety

This study will utilize the CTCAE Version 4.03 for adverse event reporting. TTC-related toxicities are defined as toxicities that occur after SBRT treatment start and that are judged, by the sponsor or investigators, to be definitely, probably or possibly related to the combination of dual immunotherapy and SBRT based on the location of the metastases treated as well as exposure of surrounding normal tissues. Toxicity that is clearly and directly related to the primary disease or to another etiology is excluded from this definition. The following toxicities related to TTC are expected:

- Any Grade 4 immune-related AE (irAE)
- Any ≥ Grade 3 colitis
- Any Grade 3 or 4 non-infectious pneumonitis irrespective of duration
- Any Grade 2 pneumonitis that does not resolve to ≤ Grade 1 within 3 days of the initiation of maximal supportive care
- Any Grade 3 irAE, excluding colitis or pneumonitis, that does not downgrade to Grade 2 within 3 days after onset of the event despite optimal medical management including systemic corticosteroids or does not downgrade to ≤ Grade 1 or baseline within 14 days
- Liver transaminase elevation > 8 × ULN or total bilirubin > 5 × ULN
- Grade 3 or 4 Pericarditis
- Grade 3 or 4 Esophagitis
- Grade 3 or 4 Central Airway/Bronchial Injury
- Grade 3 or 4 Radiation pneumonitis
- Grade 3 or 4 Radiation-induced liver disease
- Radiation Myelitis
- Grade 3 or 4 radiation Laryngitis or Pharyngitis
- Any other ≥ Grade 3 non-irAE, except for the exclusions listed below

The expected TTC-related toxicity definition defined above excludes the following conditions:

- Grade 3 fatigue lasting ≤7 days
- Grade 3 endocrine disorder (thyroid, pituitary, and/or adrenal insufficiency) that is managed with or without systemic corticosteroid therapy and/or hormone replacement therapy and the subject is asymptomatic
- Grade 3 inflammatory reaction attributed to a local antitumor response (eg. inflammatory reaction at sites of metastatic disease, lymph nodes, etc.)
- Concurrent vitiligo or alopecia of any AE grade
- Grade 3 infusion-related reaction (first occurrence and in the absence of steroid prophylaxis) that resolves within 6 h with appropriate clinical management
- Grade 3 or 4 neutropenia that is not associated with fever or systemic infection that improves by at least 1 grade within 3 days. Grade 3 or Grade 4 febrile neutropenia will be a SAE regardless of duration or reversibility
- Grade 3 or 4 lymphopenia
- Grade 3 thrombocytopenia that is not associated with clinically significant bleeding that requires medical intervention, and improves by at least 1 grade within 3 days
- Isolated Grade 3 electrolyte abnormalities that are not associated with clinical signs or symptoms and are reversed with appropriate maximal medical intervention within 3 days.

#### Adverse event reporting

All serious adverse events (SAE) defined as per ICH guidelines (see below) and other adverse events will be recorded on case report forms. In addition, all serious adverse events and adverse event of special interests will be reported by using the SAE form and will be submitted to Ozmosis Research Inc. within 24 h.

##### Responsibility for reporting serious adverse events to Health Canada

Ozmosis Research Inc. will provide expedited reports of SAEs to Health Canada according to applicable guidelines and regulations (including the 7-day notification for death and life-threatening events), i.e. events which are BOTH serious AND unexpected, AND which are thought to be related to protocol treatment (or for which a causal relationship with protocol treatment cannot be ruled out). Non-serious adverse event of special interests will not be reported to Health Canada.

##### Responsibility for reporting serious adverse events to drug manufacturer

Ozmosis Research Inc. will be responsible for submitting SAE (Initial and/or Follow-up reports) to AstraZeneca/MedImmune using the Ozmosis SAE form. The SAE form must be faxed to (AstraZeneca/MedImmune) at the latest by 15 days after Ozmosis is made aware of the SAE. The foregoing is applicable to all SAEs, irrespective of causality. Adverse event of special interests must be submitted to AstraZeneca/MedImmune within 15 days of Ozmosis becoming aware of the event.

##### Reporting serious adverse events to local research ethics boards

Ozmosis Research Inc. will notify all Investigators of all Serious Adverse Events that are reportable to regulatory authorities in Canada from this trial or from other clinical trials as reported to Sponsor. This includes all serious events that are unexpected and related to protocol treatment. Investigators must notify their Research Ethics Boards (REBs) and file the report with their Investigator Site File. Documentation that serious adverse events (SAEs) have been reported to REBs must be kept on file at Ozmosis Research Inc.

Documentation can be any of the following:

- letter from the REB acknowledging receipt
- stamp from the REB, signed and dated by REB chair, acknowledging receipt
- letter demonstrating the SAE was sent to the board

All expedited serious adverse events occurring within a centre will also be reported to local REBs. All adverse event of special interests will be reported as per local ethics guidelines.

The SAE reporting period begins on Cycle 1 Day 1 of study drug administration and ends 90 days after study drug discontinuation. For SAEs that have been deemed by the investigator as at least possibly related to protocol treatment, the SAE must be reported even if this occurs after the 90 days after study drug discontinuation. All SAEs must be followed until resolved or become chronic/stable unless the subject is lost to follow up. Resolution status of each event should be documented in the CRF.

#### Response rate and disease progression assessment

Subjects must have measurable disease at screening and will be evaluated for response on the basis of RECIST version 1.1 criteria. Tumor measurements using physical examination, CT scan and/or MRI or other appropriate techniques deemed suitable by the investigator will be performed at screening within 45 days of subject registration and repeated at the end of every 2 cycles. In case of partial or complete response, tumor measurements will be repeated after a minimum of 4 to 6 weeks for confirmation. The overall response rate of the patients’ lesions will be based on global response of non-SBRT lesions. However, the size changes of individual lesion (required for RECIST) could be used evaluate response on a per lesion basis in post-hoc analyses.

#### Patient reported outcomes (PRO)

The EORTC QLQ-C30 (version 3.0) and head and neck cancer specific module EORTC QLQ-H&N35 will be used to assess health-related PRO [14].

#### Correlative studies

Correlative studies will consist of 1) evaluating the responses with tumor PD-L1 expressions, 2) examining the peripheral T-cell receptor repertoire and T-cell activity as predictive biomarkers, and 3) assessing the value of biometrics in prognostication and monitoring patient toxicities to treatments. Using the Ventana SP263 assay, tumors that have 25% or more tumor cells expressing PD-L1 (TC ≥ 25%) will be considered to have high PD-L1 expression; tumors with less than 25% tumor cells expressing PD-L1 will be considered to have low PD-L1 expression. Like in CONDOR study, exploratory analysis will be performed for PD-L1 at additional cutoffs of TC ≥ 1% and TC ≥ 10% [54].

#### Follow-up procedures

Follow up for survival status and subsequent anti-cancer therapy will occur every 3 months from the time of treatment discontinuation. Radiology will be performed during follow up as per standard of care for patients that go off treatment without radiologic progression. All patients will be followed until death or up to a maximum of 2 years. Patients who are permanently discontinued from receiving protocol treatment will be followed for safety as per the protocol specified AE/SAE reporting period. All patients will be followed for survival. Patients who decline to return to the site for evaluation will be offered follow-up by phone every 3 months as an alternative. Patients who withdraw consent will not receive any further study treatment or observation, with the exception of follow-up for survival, unless the patient has expressly withdrawn consent for this.

#### Data safety monitoring board

The Data Safety Monitoring Board (DSMB) will consist of one radiation oncologist experienced in SBRT and one medical oncologist experienced in administering immunotherapies. The DSMB will meet every 6 months commencing after the first patient enrollment. If required, the DSMB will meet more frequently to review safety data per their discretion. The DSMB is independent from the sponsor and competing interests. The sponsor and Ozmosis Research will have copies of the DSMB charter.

### Statistics

#### Analysis set

##### Full analysis set (FAS)

All subjects who provide informed consent, and are screened and found eligible to enter the study, or who receive any amount of study treatment, will belong to the FAS population. This population will be used for descriptive analysis.

##### Safety analysis set

The safety population will include all FAS subjects who received at least one dose of study treatment (complete or partial, either durvalumab, tremelimumab or SBRT or any combination). This population will be used in the evaluation of safety and tolerability.

##### Evaluable set

This is the primary analysis set for this study, and includes all enrolled patients who were eligible for the study, received the combination of treatment (durvalumab, tremlimumab and SBRT in at least one lesion during the first 2 cycles), and either experienced TTC-related toxicity within 6 weeks from the start of SBRT treatments, or completed the 6 week period.

##### Abscopal rate set

The abscopal rate set will include any patient within the evaluable set who did not receive SBRT to at least one other metastatic lesion. This population will be used to assess the rate of abscopal events from the combination therapy.

#### Statistical analyses

There is no formal interim analysis specified by the protocol. Ad-hoc analyses as requested by the sponsor (e.g. abstracts, posters, oral presentations, etc.) may be performed as needed regardless of monitoring or data management status.

In the Phase I component (first 12 evaluable patients) if more than 4 patients (33%) develop ≥1 grade 3–5 TTC-related toxicity within 6 weeks from the start of SBRT, the treatment combination would be considered toxic and the study will be discontinued.

##### Analysis of phase I primary safety endpoint (TTC-related toxicity)

The analysis of the TTC-related toxicity will be based on the evaluable set, and will describe TTC-related SAE occurring within 6 weeks of SBRT start. Due to the small sample size, data will be summarized as frequencies and percentages. Exact confidence intervals, if presented, will have a 95% confidence level.

##### Analysis of phase II primary efficacy endpoint (PFS)

The PFS will be evaluated using the Kaplan-Meier method. For subjects who are alive and progression-free at the time of data cutoff for analysis, PFS will be censored at the last tumor assessment date. PFS of all treated lesions will be estimated using the Kaplan Meier method.

##### Analysis of secondary endpoints

The number and percentage of subjects reporting treatment-associated AEs will be summarized overall, and by the worst CTCAE grade and system organ class, and at each time point (prior to radiotherapy and at 3, 6, 12 and 28 weeks post-radiotherapy). In addition, the number and percentage of subjects reporting TTC-related AEs considered to be definitely, probably or possibly related to the combination of treatments, beyond what is expected by either treatment alone will be summarized at each time point. The LC and OS will be evaluated using the Kaplan-Meier method. As abscopal effect cannot be distinguished from systemic effect of immunotherapy, it will therefore be evaluated descriptively, and will be based on the abscopal rate set. Mixed model analysis will be used to assess PRO changes over time; a change of at least 10 points on any quality of life scales is defined as being clinically significant.

#### Sample size

##### Phase I

Sample size is based on feasibility and a total of 12 evaluable subjects are planned. If some subjects withdraw from the study or discontinue the study for reasons other than TTC-related toxicities within 6 weeks of SBRT start, then additional subjects (i.e. replacement subjects) may be enrolled to keep the number of subjects evaluable for the primary endpoint, determined by the rate of TTC-related SAE occurring within 6 weeks of SBRT start. Considering that discontinuation of tremelimumab and durvalumab due to toxicity was reported to be 28% [34], we estimate that a maximum of 16 subjects will be enrolled to ensure 12 evaluable patients reaching SBRT treatment between cycles 2 and 3.

The proportion of toxicity event is expected to be 10 to 15%; 12 evaluable patients would provide 44.3 -65.9% power to exclude a TTC-related adverse event rate > 34%, based on one-sided exact binomial test. Additionally, the upper bounds around the adverse event rates will be 26.5% or 38.5% if the observed number of adverse events in the study is 0 or 1 respectively.

For 12 subjects, power to exclude a TTC-related event rate > 34% will vary according to the proportion of toxicity expected (Table 3). Lung, liver and spine are the most common sites treated with SBRT (extracranial). Rates of grade 3 toxicity is 3% with liver SBRT [55], 8% in spine SBRT [56] and less than 5% in lung SBRT [57, 58]. Therefore, it is reasonable to expect a rate of TTC-related toxicity to be within 10–15%.

**Table 3:** TTC-related event rate > 34% according to proportion of toxicity

Expected overall adverse event (Proportion)	Adverse event rate (Null proportion)	Lower critical value	Power
5%	34%	1	88.2%
10%	34%	1	65.9%
15%	34%	1	44.3%
20%	34%	1	27.5%

##### Phase II

For the phase 2 (efficacy) component, the study will provide an estimate of PFS rate at 6-month. Sample size is based on feasibility and a total of 35 evaluable subjects are planned. Additional subjects (ie. replacement subjects) may be enrolled to keep the number of subjects evaluable for the primary endpoint, determined by the rate of TTC-related SAE occurring within 6 weeks of SBRT start. Considering that discontinuation of tremelimumab and durvalumab due to toxicity was reported to be 28% [34], we estimate that a maximum of 45 subjects will be enrolled to ensure 35 evaluable patients. In the absence of prospective data of the combined effects of SBRT with durvalumab and tremelimumab in the treatment of HNSCC metastases, we hope that the study could provide an estimate of PFS rate at 6-month and based on the estimate we will decide whether to conduct further research for this combination. In CheckMate 141 [25] nivolumab was associated with a 6-month PFS of 20% compared to 10% with standard chemotherapy in patients with platinum-refractory recurrent and metastatic HNSCC. Considering that the current trial will include patients that have failed nivolumab alone or nivolumab plus durvalumab, an observed PFS rate of 27% at 6-month with a sample size of 35 patients would provide 95% two sided confidence intervals between 12 and 43% of the 6-month PFS.

### Discontinuation, replacement of subjects and retreatment

#### Permanent discontinuation of durvalumab, tremelimumab

An individual subject will not receive any further investigational product if any of the following occur in the subject in question:

1. Subject weight falls to 30 kg or less
2. Withdrawal of consent or lost to follow-up
3. Adverse event that, in the opinion of the investigator or the sponsor, contraindicates further dosing
4. Subject is determined to have met one or more of the exclusion criteria for study participation at study entry and continuing investigational therapy might constitute a safety risk.
5. Pregnancy or intent to become pregnant
6. Any AE that meets criteria for discontinuation as defined in Section 6.4.
7. Subject noncompliance that warrants withdrawal.
8. Initiation of alternative anticancer therapy including another investigational agent
9. Confirmation of PD and investigator determination that the subject is no longer benefiting from treatment with durvalumab + tremelimumab.
10. Inability to reduce corticosteroid to a dose of ≤10 mg of prednisone per day (or equivalent) within 12 weeks after last dose of study drug/study regimen

Subjects who are permanently discontinued from receiving investigational product will be followed for safety unless consent is withdrawn or the subject is lost to follow-up or enrolled in another clinical study. All subjects will be followed for survival. Subjects who decline to return to the site for evaluations will be offered follow-up by phone every 3 months as an alternative.

#### Replacement of subjects

Subjects will be replaced in the following circumstances:

- Withdrawal or permanent discontinuation from the study ≤6 weeks from SBRT start or before SBRT start due to reasons other than TTC-related toxicities.
- Subject death before SBRT start.

#### Retreatment

For patients who develop a response on durvalumab and tremelimumab combination (during the first 4 cycles of treatment) and later develop a PD on durvalumab alone, the investigator will restart durvalumab and tremelimumab combination if the patient does not have any significant, unacceptable or irreversible toxicities.

### Follow-up following study treatment discontinuation

For the SAEs that have been deemed by the investigator as unrelated to protocol treatment, the SAE reporting period begins after Cycle 1 Day 1 of durvalumab + tremelimumab dose administration and ends 90 days after discontinuation of the study drug. For the SAEs that have been deemed by the investigator as at least possibly related to protocol treatment, the SAE must be reported even if it occurs after 90 days after discontinuation of the study drug. The investigator and/or Sponsor are responsible for informing their Ethics Committee of the SAE as per local requirements. During the course of the study all AEs and SAEs will be proactively followed up for each subject.

### Auditing

Authorized representatives of MedImmune, a regulatory authority, or an IRB/IEC may perform audits or inspections at the center, including source data verification.

### Amendments

All protocol amendments will be confirmed in writing and submitted, as appropriate, for review by the REB. Amendments will be reviewed and approved by REBs prior to local implementation, EXCEPT when the amendment eliminates an immediate hazard to clinical trial patients or when the change(s) involves only logistical or administrative aspects of the trial.

### Consent and confidentiality

All enrolled patients will be required to sign informed consent before study entry. Consents will be obtained by designated research assistants. Confidentiality of the information collected will be respected at all time. The principal investigator, the co-investigators and the research nurses will gather and record all collected information in a research records. All information collected will remain strictly confidential to the extent permitted by law. In all research records, subjects will be identified by enrolment number.

### Results dissemination

Results of the phase I and the phase II of the study will be disseminated in minimally 2 publications, regardless of the study outcomes. Results of the trial will also be submitted to the ClinicalTrials.gov database, where this study is registered.

## Discussion

Patients with metastatic HNSCC typically have a dismal prognosis and limited systemic treatment options. In recent years, oncology practise has shifted towards aggressive treatment of oligometastatic cancers, with the underlying rationale that radical ablation of limited disease burden can lead to prolonged PFS and sometimes potentially achieve cure. The high immunogenicity of HNSCC as well as the prolonged survival and good response rates observed in recurrent and metastatic HNSCC with use of anti-PD1 therapy supports the benefit of immunotherapy to improve outcomes in this patient population. In addition, the clinical activity of the combination of durvalumab and tremelimumab noted in study by Antonia et al. in patients with PD-L1-negative tumours supports the increased efficacy of this combination [34].

Pre-clinical and clinical [48, 51–53, 59] data suggests that the local use of SBRT can generate an immune response, notably PD-L1 up-expression and abscopal effect on non-irradiated tumor sites. A phenomenon of antigen release with use of high dose per fraction (10–15 Gy) was shown in a study by Golden et al., suggesting that SBRT may work synergistically with immunotherapy [50]. Furthermore, as Huang et al. suggested that lower tumor burden is associated with higher response rates to immune checkpoint inhibition [60], maximal reduction of disease burden using SBRT in oligometastatic diseases may improve treatment efficacy. SBRT combination with dual inhibition of both PD-1/PD-L1 and CTLA-4 pathways therefore holds the promise to significantly potentiate treatment effect, to increase treatment response rate as well as to prolong PFS and OS. Dual checkpoint blockade in combination with radiotherapy has not yet been reported in human clinical trials. This study will therefore constitute the first prospective data of the combined effects of SBRT with dual checkpoint blockade with durvalumab and tremelimumab in the treatment of HNSCC metastases. The study will determine the safety of this TTC and will provide an estimate of PFS rate at 6-month. In the event of acceptable toxicity and promising PFS, results of this study would lead to a phase III trial testing this treatment combination against standard of care in metastatic HNSCC.

## Abbreviations

AE: Adverse events
CT: Computed tomography
CTCAE: Common Terminology Criteria for Adverse Events
EORTC: European Organization for Research and Treatment of Cancer
FAS: Full analysis set
GTV: Gross tumor volume
HNSCC: Head and neck squamous cell carcinoma
ILD: Interstitial lung disease
IP: Investigational product
IrAE: Immune-related AE
LC: Local control
LN: Lymph nodes
OS: Overall survival
PFS: Progression free survival
PRO: Patient reported outcomes
PTV: Planning treatment volume
Q4W: Every 4 weeks
QLQ-C30: Quality of Life Questionnaire Core-30
SAE: Serious adverse events
SBRT: Stereotactic Body Radiotherapy
TTC: Triple treatment combination
